# Psychiatric Assessment in Patients with Mild Temporal Lobe Epilepsy

**DOI:** 10.1155/2019/4139404

**Published:** 2019-01-14

**Authors:** Antonella Bruni, Iolanda Martino, Maria Eugenia Caligiuri, Maria Grazia Vaccaro, Michele Trimboli, Cristina Segura Garcia, Pasquale De Fazio, Antonio Gambardella, Angelo Labate

**Affiliations:** ^1^Neurology Unit, Department of Medical and Surgical Sciences, University Magna Graecia, Catanzaro, Italy; ^2^Psychiatric Unit, Department of Health Sciences, University Magna Graecia, Catanzaro, Italy; ^3^Neuroscience Research Center, University Magna Graecia, Catanzaro, Italy

## Abstract

**Objectives:**

The findings of previous studies focused on personality disorders in epileptic patients are difficult to interpret due to nonhomogeneous samples and noncomparable methods. Here, we aimed at studying the personality profile in patients with mild temporal lobe epilepsy (mTLE) with psychiatric comorbidity.

**Materials and Methods:**

Thirty-five patients with mTLE (22 males, mean age 40.7 ± 12.1) underwent awake and sleep EEG, 3T brain MRI, and an extensive standardized diagnostic neuropsychiatric battery: Temperament and Character Inventory-Revised (TCI-R), Beck Depression Inventory-2, and State-Trait Anxiety Inventory. Drug history was collected in detail. Hierarchical Cluster Analysis was performed on TCI-R data, while all other clinical and psychological variables were compared across the resulting clusters.

**Results:**

Scores of Harm Avoidance (HA), Reward Dependence (RD), Persistence (P), Cooperativeness (C), and Self-Transcendence (ST) allowed the identification of two clusters, describing different personality subtypes. Cluster 1 was characterized by an early onset, more severe anxiety traits, and combined drug therapy (antiepileptic drug and Benzodiazepine/Selective Serotonin Reuptake Inhibitors) compared to Cluster 2.

**Conclusions:**

Our findings suggest that different personality traits may play a role in determining the clinical outcome in patients with mTLE. Specifically, lower scores of HA, RD, P, C, and ST were associated with worse clinical outcome. Thus, personality assessment could serve as an early indicator of greater disease severity, improving the management of mTLE.

## 1. Introduction

Temporal lobe epilepsy (TLE), the most common type of focal epilepsy in adulthood, is frequently associated with psychiatric disorders [1, 2], which mainly occur in the form of depression and generalized anxiety disorders [3].

Over the past 2 decades, studies from nonsurgical series of mesial TLE patients confirmed the existence of a mild form of mesial TLE (mTLE), which is characterized by at least 24 seizure-free months with or without antiepileptic medication [4].

Early diagnosis—and consequent management—of psychiatric symptoms in TLE patients is desirable, since it might lead to a better epilepsy outcome, both in terms of quality of life and of healthcare costs [5]. Recently, the presence of psychiatric comorbidities has been deeply investigated in patients with refractory TLE, especially with the aim of improving the identification of optimal candidates for surgery. In fact, while the success rate of resection of epileptogenic tissue is increasing, thanks to advances in neurosurgical techniques, up to 40% of patients still suffer disabling seizures after intervention [6]. In this context, it is possible that underrecognized—and thus undertreated—psychiatric comorbidities may influence not only seizure control and tolerance to medications but also surgical outcome. It has been also suggested that the quality of life of epileptic patients may be influenced by personality traits [7] and that, in turn, these traits might be negatively affected by the presence of depression and anxiety [8]. Thus, the personality assessment may be a useful tool for reducing negative effects on perceived social support and quality of life in TLE. The prognostic potential of this tool for epilepsy outcome in patients with TLE should be investigated not only in syndromes associated with drug-resistance but also in a population of patients in which seizures are well controlled such as mTLE [9–11]. A quantitative and validated instrument to study the expression of personality traits is the psychobiological model of Cloninger, known as the Temperament and Character Inventory-Revised (TCI-R), which provides a distinction between individual subtypes of personality [12]. In this study, we aimed to investigate the personality profile in a population of consecutive patients with mTLE.

## 2. Materials and Methods

### 2.1. Participants

Patients were enrolled from the outpatient clinic of a tertiary epilepsy center, the Institute of Neurology, and from the Unit of Psychiatry of Magna Graecia University, Catanzaro. All subjects gave informed consent to participate. The study was approved by the local ethics committee and was conducted in accordance with the Helsinki Declaration. The study group consisted of 58 consecutive patients with mTLE (mean age 40.7 ± 12.1). Diagnosis of epilepsy was assessed by trained epileptologists (AL and AG), based on the ILAE International Classification of Epilepsies [13]. The following demographic and clinical characteristics were collected: sex, age, family history of epilepsy and febrile convulsions, age at onset of seizures, seizure-free period, use of Antiepileptic Drugs (AEDs), and psychiatric treatment. Participants underwent routine awake and sleep electroencephalograms (EEGs). The interictal EEGs were recorded according to the 10-20 international system with supplementary electrodes over the temporal lobe. Finally, all patients underwent a 3T brain MRI protocol (Discovery MR-750, General Electric, Milwaukee, WI), optimized for epilepsy purposes [14, 15]. Psychiatric standard assessment was conducted by qualified psychiatrists (AB, PDF, and CSG) through the Diagnostic and Statistical Manual for Psychiatric Disorders-5th Edition (DSM-5) [16]. Two psychologists (IM and MGV) performed a comprehensive psychometric evaluation. The severity of depression symptoms was assessed through the Beck Depression Inventory-2 (BDI), a self-report questionnaire with 21 items, rated on a 4-point scale from 0 to 3 [17]. The assessment of anxiety symptomatology was performed with the State-Trait Anxiety Inventory (STAI Y1 and Y2) [18]. This self-administered scale clearly differentiates between the temporary condition of state anxiety and the more general and long-standing trait anxiety. There are in fact two separate scales each having 20 items. Both parts of the scale can be applied at the same time, with the STAI-state completed first. The total score obtained from both subscales on the STAI ranges from 20 to 80. A higher score indicates higher levels of anxiety. Inclusion criteria for participants were the following: (1) diagnosis of mTLE [10], (2) age range 18-70, (3) MMSE > 26, and (4) consent to formal psychiatric evaluation. Thirty-five subjects (age range 18-65 y) met inclusion criteria, whereas 13 patients were excluded because they had not completed the instrumental evaluation or the battery of psychological tests.

### 2.2. Personality Assessment

The dimensional approach was assessed using Cloninger's TCI-R, which consists of 240 items that evaluate temperament and character [19]. The four dimensions of temperament are Novelty Seeking (NS, i.e., tendency towards excitement in response to novel or rewarding stimuli), Harm Avoidance (HA; tendency to respond intensively to adverse stimuli), Reward Dependence (RD; tendency to respond intensively to reward signals and to maintain behavior previously associated with reward), and Persistence (P; ability of resisting frustration). The three dimensions of character are Self-Directedness (SD), Cooperativeness (C), and Self-Transcendence (ST), based on the concept of “myself, others, and world” and representing purposes, goals, and attitude, respectively, of the individual. All items are measured on a 5-point Likert scale. The TCI-R shows good internal consistency (range 0.76-0.89).

### 2.3. Hierarchical Cluster Analysis

In order to identify subtypes of personality in a *data-driven* way, i.e., without any a priori hypothesis, we performed a hierarchical agglomerative Cluster Analysis based on the personality assessment. In particular, the scores and subscores of NS, HA, RD, P, SD, C, and ST of all mTLE patients were fed to the algorithm. Hierarchical Cluster Analysis initially considers each patient as a single cluster (i.e., group) and then iteratively combines pairs of most “similar” clusters until a single one (i.e., the entire cohort) is left [20]. The result is a graphical representation called a *dendrogram*, which allows the choice of the number of clusters to consider. The similarity between different subjects, which is needed to define the clusters, was measured by Ward's clustering linkage criterion: at each step, the pair of clusters with the minimum sum of square errors is merged into a single cluster. Unlike in regression modeling, Cluster Analysis does not involve parameter estimation, which makes it less prone to overfitting (caused by consideration of too many variables in a small sample size).

### 2.4. Statistical Analysis

After Cluster Analysis was performed on the basis of TCI-R dimensions only, the number of identified groups was selected from the dendrogram. Cluster-wise significant differences in all other clinical variables were evaluated by pairwise *t*-tests. Significance level was set to 0.05 after Bonferroni correction for multiple comparisons.

## 3. Results

**Figure 1:**
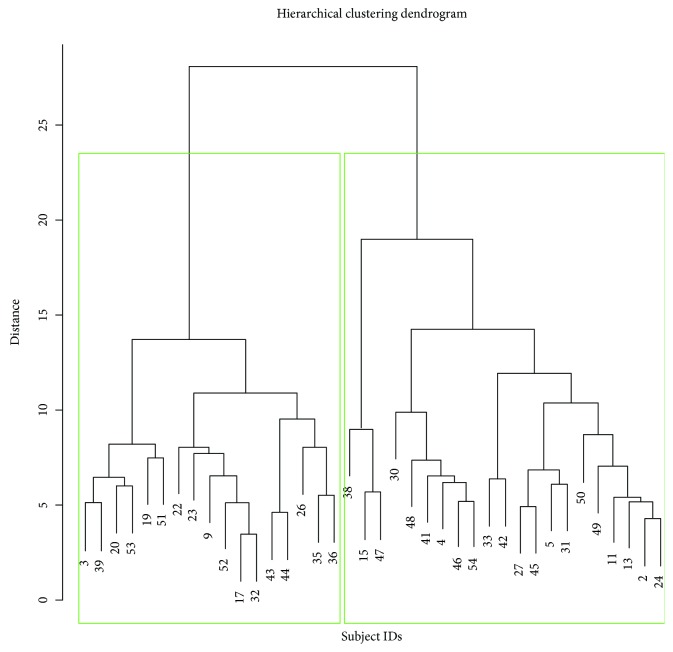
Graphical representation of hierarchical clustering results. Based on the dendrogram, we considered two personality clusters, highlighted by green boxes.

**Figure 2:**
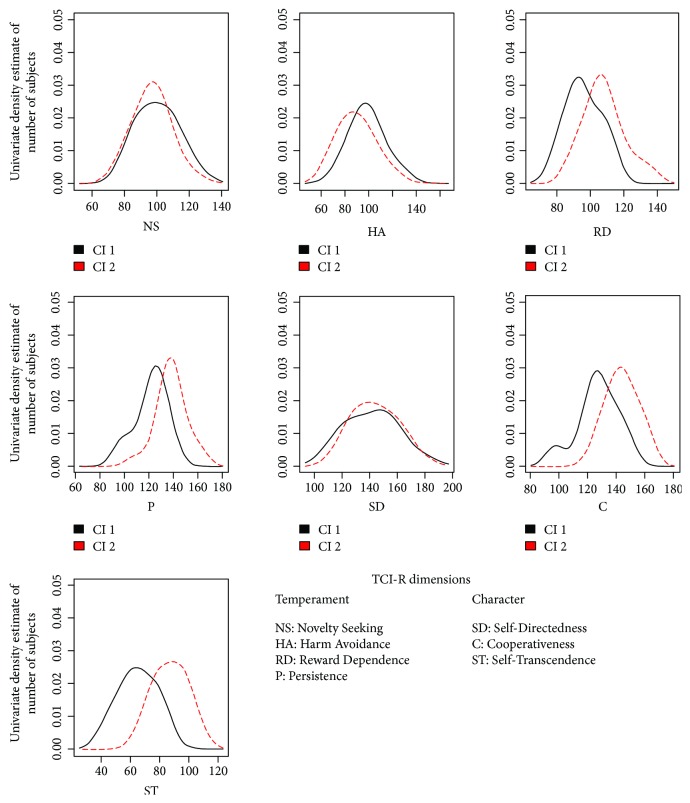
Distribution of TCI-R scores across clusters. Abbreviations: Cl 1: Cluster1; Cl 2: Cluster2; TCI-R: Cloninger's Temperament and Character Inventory-Revised.

**Figure 3:**
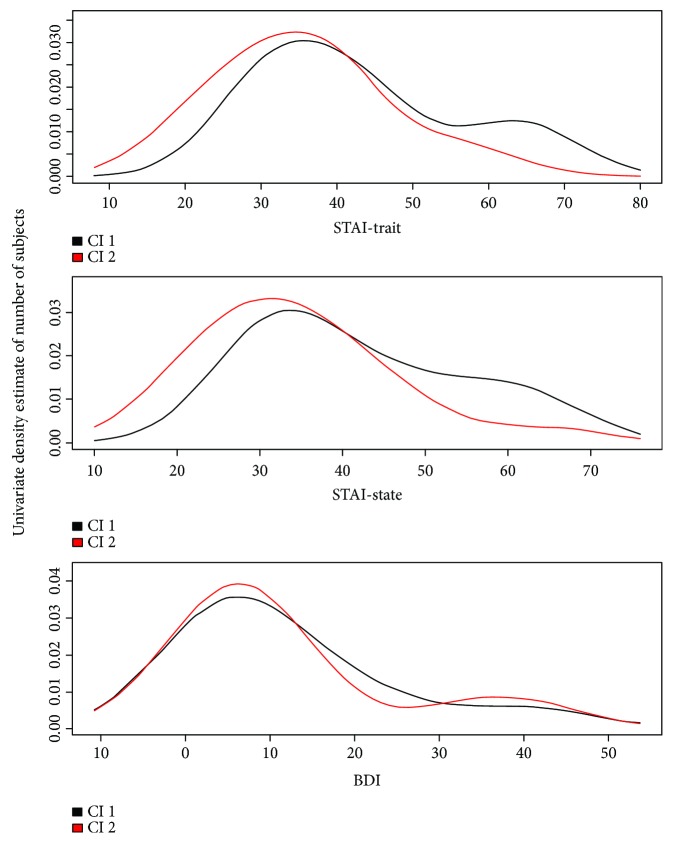
Distribution of STAI and BDI scores across clusters. Abbreviations: Cl 1: Cluster1; Cl 2: Cluster2; STAI: State-Trait Anxiety Inventory; BDI: Beck Depression Inventory.

All patients had diagnosis of mTLE and uniform psychopathological pattern. The demographic, clinical, and psychological features of the entire cohort are summarized in Table 1. Levels of depression (BDI: 11.94 ± 12.38 [cut-off > 12]) and anxiety (STAI Y1: 37.40 ± 12.46; STAI Y2: 37.37 ± 12.54 [STAI Y1-Y2, cut-off > 40]) were not high. None of the patients met criteria for a major depressive disorder or generalized anxiety disorder, according to DSM-5. The dendrogram produced by the Hierarchical Cluster Analysis of TCI-R scores and subscores is shown in Figure 1. Two clusters (Cluster 1 and Cluster 2) were selected, and their characteristics were compared. Clinical features and mean scores of TCI-R dimensions and subfacets for Cluster 1 and Cluster 2 are shown in Table 2. There were no significant differences in disease duration, interictal EEG features, and number of experienced auras. TCI-R scores of the two clusters were compared to the mean value of the general population. Independently from age and sex, we were able to identify two personality subtypes, characterized by different levels on several TCI-R dimensions (Figure 2, Table 2): Cluster 1 had significantly lower scores on RD (in particular its subfacets “sentimentality” and “openness to warm communication”), P (and its subfacet “perfectionist”), and ST (along with its subfacets “self-forgetful” and “transpersonal identification”). Focusing on TCI-R subscales only, significant differences between the two clusters were also observed for “impulsiveness” (second subfacet of NS) and “purposeful” (second subfacet of SD). Cluster 1 was also characterized by higher levels on HA, albeit not reaching significance. Clinically, patients included in Cluster 1 had an earlier age at onset and had a more complex disease, as can be seen in Table 3: eight out of 18 patients in Cluster 1 (44%) were taking psychiatric therapy—three took Benzodiazepine (BDZ; two Alprazolam 0.50 mg/die and one Lorazepam 1 mg/die), one took a Selective Serotonin Reuptake Inhibitor (SSRI; Fluoxetine 50 mg/die), two took a combination of BDZ and SSRI (Lorazepam 1 mg/die and Paroxetine 20 mg/die), and two took Neuroleptics (NL; Haloperidol 2 mg/die and Risperidone 2 mg/die). Only one out of 17 patients (5.8%) in Cluster 2 was taking Alprazolam (0.25 mg/die). STAI and BDI scores across the two clusters are shown in Figure 3: bimodal patterns can be observed, and for what concerns the STAI scores, patients with higher anxiety were all assigned to Cluster 1.

## 4. Discussion

In this study, we evaluated the potential of personality assessment as a prognostic indicator of clinical outcome in mTLE patients. Although 58 patients were recruited, only 35 patients were able to complete the entire battery, which includes the 240 Cloninger's items. However, we were aware of potential dropout in our study because this phenomenon is very frequent when the psychiatric battery is complex. This strict selection was made in order to improve the value of our work. By using Cloninger's TCI-R, we were able to identify two distinct personality profiles in this population with mild epilepsy, which showed differences on the following TCI-R scores: Reward Dependence, Persistence, Cooperativeness, and Self-Transcendence (all lower in Cluster 1) and Harm Avoidance (higher in Cluster 1). Interestingly, these personality facets seemed associated with a worse clinical outcome in mTLE patients assigned to Cluster 1.

Patients with refractory TLE, in which personality traits might already be altered by seizure recurrence, have been the focus of the majority of studies [7, 21]. However, when trying to assess the prognostic value of personality assessment in epilepsy, it is also important to consider a population in which seizures are well controlled and do not influence the perceived quality of life [9]. In fact, some authors found clinically significant obsessive symptoms in patients with TLE and idiopathic generalized epilepsy, suggesting that temporal lobe involvement may play a role in the development of specific psychopathological syndromes [22]. Conversely, in our sample we have identified different psychopathological domains; however, typical traits of obsessive personalities or C clusters (such as Perfectionism) have emerged (Table 3).

Mild TLE represents a precious resource to study the correlates underlying the epileptic syndrome itself, since it is defined by relatively mild clinical features [10]. Our group recently demonstrated that almost 25% of mTLE patients might become refractory at a very long-term follow-up [11]. In this previous study, we had found that higher age at disease onset, presence of hippocampal sclerosis, and history of febrile convulsions were risk factors for changing disease course. To complete the multidomain assessment of these demographic and radiological risk factors, it is reasonable to hypothesize that psychometrics also may influence a patient's long-term epilepsy outcome—as happens for psychiatric comorbidities, which may hamper seizure control after surgery [6]. Different from the notable study by Bear and Fedio, our sample of mTLE did not show significant differences with respect to the side of the lesion, which was analyzed in Table 3 [23]. Despite several studies having investigated personality disorders in TLE, it is difficult to characterize a typical personality profile of these patients, due to the presence of psychiatric comorbidities that could in turn negatively influence personality [8]. Here, we performed a thorough psychiatric screening to limit confounding effects of severe depression and/or anxiety. Results from existing studies may be difficult to compare, due to the variety of diagnostic tools used [20, 21]. For this reason, we assessed personality using Cloninger's TCI-R, a neurobiological self-report questionnaire for identification of personality subtypes. Cloninger described seven dimensions of personality: four about temperament (Novelty Seeking, Harm Avoidance, Reward Dependence, and Persistence) and three about character (Self-Directedness, Cooperativeness, and Self-Transcendence). Character refers to a cognitive process including intensions and attitudes. Temperament determines mood and behavior with subcortical elaboration [19]. Cluster Analysis on temperament and character scores allowed us to identify a subgroup of patients with mTLE (Cluster 1) with potentially dysfunctional personality traits: compared to Cluster 2, Cluster 1 had lower scores on Reward Dependence, Persistence, Cooperativeness, and Self-Transcendence, while having higher scores on Harm Avoidance. Of note, in Cluster 1 the social interaction temperament variables were lower (i.e., Reward Dependence and Persistence), more closely related to associative conditioning learning (unconscious automatic reactions). This suggests that these patients tend to be more anxious; suspicious; less persistent; uninterested in social interactions, with lack of empathy; and sometimes hypochondriac.

Thus, we can speculate that the personality profile of Cluster 1 might justify the poor compliance to treatment of some TLE patients, who might also experience difficulties in building an empathic relationship with the clinician. If so, early identification of these personality traits might be useful in enhancing patient care, especially for those who tend to request several opinions from different specialists and take combined therapies. Despite TCI-R-altered values, scores in Cluster 1 defined only dysfunctional traits, but not a structured personality disorder (e.g., borderline). A previous study found personality changes in epilepsy patients according to Cloninger's model, suggesting a relationship between epilepsy and psychiatric comorbidity, but did not clarify the role of depression and anxiety [24]. It has also been suggested that specific traits could improve the choice of the optimal AED [25]. This emphasizes the importance of understanding a patient's attitude, so that the treatment outcome is not compromised by factors independent from efficacy or side effects. The link between psychological factors and compliance to epilepsy treatment has been investigated considering depression, anxiety, low self-esteem, stigma, discrimination, and adverse physical changes [26–30]. Some authors found a reciprocal influence between epilepsy and mood/anxiety comorbidities [29]. A previous study has demonstrated that patients with TLE suffer from problems in communication and interpersonal relations; uncertainties and controversies remain as to the precise goings-on of these psychological difficulties [31]. Broicher et al. comparing MTLE patients with extra-MTLE patients identified MTLE as a significant risk factor for the development of deficits in social cognition empathizing the stigma associated with epilepsy [31]. This aspect has been already described within the “interictal personality syndrome” and the “Waxman-Geschwind syndrome” [23, 32].

Others author argued against a specific TLE personality trait and suggested that personality features may be related to the experience of repetitive seizures, rather than the specific underlying pathophysiology of TLE [23, 31, 32].

The role of personality in affecting the course of epilepsy, therefore, has never been clearly delineated. Our study highlights the important role of personality in epileptic patients even in the presence of mild anxiety and depression symptoms. The use of TCI-R also allowed us to identify a specific neurobiological circuit, which will be the focus of future studies.

Few limitations need to be accounted for. First of all is the small sample of our population. It should be considered, however, that the population recruited was peculiar: it requires epileptologist experts for the diagnosis and not many patients are keen to undergo deep psychiatric evaluation. Secondly, our results represent preliminary data and are propaedeutic to a further study with a larger population including other forms of epilepsy. Thirdly, although nine patients were taking psychiatric therapy, its eventual influence seems limited, since Cluster Analysis classified 8 out of 9 of these patients in Cluster 1, independently from information on psychiatric therapy (the algorithm worked only on personality data). Moreover, low STAI and BDI scores may be influenced by AEDs, which could also play a protective role even in endogenous subjects; however, the vast majority of our sample was taking a single AED or bitherapy at a very low dosage. Psychiatric effects of AEDs on anxiety and depression (but not on personality) are recognized to have an important role in predisposing to or protecting against psychiatric disorders [25, 26]. Future works will include the analysis of MRI images in order to detect possible neuroimaging correlates associated with the personality profile.

## Figures and Tables

**Table 1:** Demographical, clinical, and psychological characteristics of the patients.

Variable	TLE (*n* = 35)
*Demographical and clinical data*	
Gender (f/m)	13f/22 m
Educational level (y)	13 (8-18)
Age (y)	40.7 ± 12.1
Age at onset (y)	25.0 ± 13.5
Duration (y)	15.1 ± 8.7
Antecedent FCs, *n*	5
Interictal EEG, *n*	
Unilateral right	8
Unilateral left	6
Bilateral	13
Normal	5
Radiological hippocampal sclerosis, *n*	
Right	5
Left	6
Bilateral	0
None	15
Aura, *n*	15
AEDs, *n*	
Monotherapy	15
Multitherapy	20
Psychiatric therapy (BDZ, SSRI, SNRI, and NL)	9
*Psychological data*	
MMSE	28.5 ± 1.5
BDI	11.94 ± 12.38
STAI—state anxiety	37.40 ± 12.46
STAI—trait anxiety	37.37 ± 12.54

Legend: BDZ: Benzodiazepine; SSRI: Selective Serotonin Reuptake Inhibitor; SNRI: Serotonin-Norepinephrine Reuptake Inhibitor; NL: Neuroleptic; MMSE: Mini-Mental State Examination (cut-off: >23); BDI: Beck Depression Inventory (cut-off: >12); STAI Y1-Y2: State-Trait Anxiety Inventory 1-2 (cut-off: >40). Data are given as mean values ± SD or median values (range) when appropriate.

**Table 2:** TCI-R scores across clusters.

TCI-R dimension and subfacets	Cluster 1 (*n* = 18)	Cluster 2 (*n* = 17)	*p* value
*NS*	100.39 ± 12.48	97.00 ± 10.80	1
Exploratory excitability (NS1)	30.67 ± 5.00	29.65 ± 5.77	1
Impulsiveness (NS2)	24.39 ± 4.19	21.59 ± 4.30	1
Extravagance (NS3)	26.56 ± 4.71	26.35 ± 4.21	1
Disorderliness (NS4)	18.78 ± 3.28	19.41 ± 3.00	1
*HA*	98.83 ± 13.89	89.88 ± 14.94	1
Anticipatory worry (HA1)	31.17 ± 6.54	28.29 ± 7.10	1
Fear of uncertainty (HA2)	24.17 ± 3.76	23.12 ± 5.34	1
Shyness (HA3)	20.83 ± 4.19	17.06 ± 5.24	0.87
Fatigability (HA4)	22.67 ± 5.38	21.41 ± 6.06	1
*RD*	96.06 ± 10.34	108.4 ± 12.25	0.10
Sentimentality (RD1)	25.72 ± 4.03	31.29 ± 4.24	**0.01**
Openness to warm communication (RD2)	34.28 ± 3.92	39.82 ± 4.81	**0.02**
Attachment (RD3)	18.17 ± 4.03	19.94 ± 5.94	1
Dependence (RD4)	17.89 ± 3.16	17.29 ± 3.65	1
*P*	121.8 ± 12.69	138.4 ± 11.95	**0.01**
Eagerness of effort (P1)	32.89 ± 5.19	35.35 ± 3.50	1
Work hardened (P2)	28.39 ± 4.94	31.94 ± 4.80	1
Ambitious (P3)	34.56 ± 3.97	39.65 ± 5.68	0.15
Perfectionist (P4)	26.00 ± 4.67	31.41 ± 3.89	**0.03**
*SD*	141.8 ± 18.55	144.5 ± 14.89	1
Responsibility (SD1)	30.72 ± 4.39	27.18 ± 5.73	1
Purposeful (SD2)	21.94 ± 2.96	24.76 ± 2.49	0.16
Resourcefulness (SD3)	17.89 ± 3.98	18.82 ± 2.72	1
Self-acceptance (SD4)	29.83 ± 8.58	30.06 ± 7.27	1
Enlightened second nature (SD5)	41.39 ± 5.62	43.65 ± 4.50	1
*C*	127.1 ± 14.27	144.1 ± 10.82	**0.01**
Social acceptance (C1)	27.72 ± 4.84	34.41 ± 4.65	**0.007**
Empathy (C2)	16.28 ± 2.59	19.53 ± 2.03	**0.009**
Helpfulness (C3)	29.00 ± 4.70	31.76 ± 2.59	1
Compassion (C4)	24.61 ± 5.16	28.59 ± 5.05	0.99
Pure-hearted conscience (C5)	29.50 ± 4.60	29.82 ± 4.40	1
*ST*	65.28 ± 12.56	87.53 ± 11.09	**0**
Self-forgetful (ST1)	23.72 ± 5.89	33.29 ± 4.87	**<0.001**
Transpersonal identification (ST2)	21.17 ± 5.53	29.76 ± 4.29	**<0.001**
Spiritual acceptance (ST3)	20.39 ± 5.42	24.47 ± 7.18	1

Legend: NS: Novelty Seeking; HA: Harm Avoidance; RD: Reward Dependence; P: Persistence; SD: Self-Directedness; C: Cooperativeness; ST: Self-Transcendence.

**Table 3:** Demographical and clinical data across clusters.

Variables	Cluster 1	Cluster 2	*p* value
Gender (f/m)	8/10	5/12	0.36
Age (y)	48 ± 4.4	52 ± 12.1	0.62
Age at onset (y)	17 ± 12.1	38 ± 22.5	0.23
Duration (y)	23.3 ± 5.8	17 ± 14.7	0.53
Antecedent FCs, *n*	4	1	0.17
Interictal EEG, *n*			
Unilateral right	3	5	0.66
Unilateral left	4	2	
Bilateral	9	7	
Normal	2	3	
Hippocampal sclerosis, *n*			
None	9	6	0.91
Right	3	2	
Left	3	3	
Aura, *n*	7	8	0.63
AEDs, *n*			
Monotherapy	6	9	0.24
Multitherapy	12	8	
Psychiatric therapy (BDZ, SSRI, SNRI, and NL)	8	1	**0.001**

## Data Availability

The data used to support the findings of this study are available from the corresponding author upon request.
